# Barriers and predictors of medication use for childhood ADHD: findings from a UK population-representative cohort

**DOI:** 10.1007/s00127-019-01720-y

**Published:** 2019-05-09

**Authors:** A. E. Russell, T. Ford, G. Russell

**Affiliations:** 1 Centre for Academic Mental Health, University of Bristol Medical School, Oakfield House, Bristol, BS8 2BN UK; 2 University of Exeter Medical School, St Luke’s Campus, Exeter, EX1 2LU UK

**Keywords:** ADHD, Gender, Pharmacology, Cohort, Epidemiology

## Abstract

**Purpose:**

Little is known about sociodemographic and clinical factors that predict and act as barriers to ADHD medication independently of symptom severity. We examined the proportion of children using medication for ADHD, age of initiation of medication, and predictors of medication use in a population-representative cohort.

**Methods:**

Data from the Millennium Cohort Study on child ADHD, medication use for ADHD at age 14 (in 2014–2015) and child, parent and sociodemographic variables were collated. Logistic regression models were used to identify factors that predict medication use for ADHD (the main outcome measure), adjusting for symptom severity at age seven.

**Results:**

The weighted prevalence of ADHD was 3.97% (*N* = 11,708). 45.57% of children with ADHD (*N* = 305) were taking medication. The median age at initiation was 9 years (range 3–14). Male gender (AOR 3.66, 95% CI 1.75, 7.66) and conduct problems at age seven (AOR 1.24 95% CI 1.04, 1.47) and 14 predicted medication use at age 14 after adjusting for symptom severity.

**Conclusions:**

Our study is the first to assess predictors of medication whist adjusting for ADHD symptom severity. Girls with ADHD were less likely to be prescribed medication, even when they displayed similar ADHD symptom levels to boys. Conduct problems also predicted medication independently of ADHD symptoms. ADHD may be more often medicated in boys because clinicians may think a prototypical ADHD child is male, and perhaps conduct problems make boys more disruptive in the classroom, leading to boys being more often treated.

## Introduction

Barriers to accessing care for attention deficit/hyperactivity disorder (ADHD) have been studied at the levels of identification and referral to specialist services [1] where an assessment is made and diagnosis assigned. There is less knowledge of whether treatment decisions are predicted by sociodemographic or clinical characteristics. Social and cultural influences impact on what treatment recommendations are made, whether children receive the most appropriate treatment [2] and also influence parents’ attitudes [3]. A recent systematic review identifies a group of “wider determinants” affecting access to care for ADHD operating at societal level including gender, age, ethnicity, socioeconomic status (SES), social networks and urban residence. Factors that predict service utilisation include comorbid disorders, adult perceptions of problems and willingness to engage [4].

Existing evidence as to whether socio-cultural factors also operate as barriers and predictors to accessing pharmacological treatment for ADHD is mixed: some studies find no differences by gender [5–7], another reports that boys are more likely to be prescribed medication [8] and one finds that treatment initiation (both pharmacological and psychotherapy) is more common in boys than girls in East Asia, although not in central Europe [9]. Co-occurring disorders, particularly conduct and oppositional disorders, have also been reported to be associated with increased likelihood of ADHD medication [5, 7, 8, 10] as have intellectual disability [8] and lower cognitive ability [7]. Low SES and being of ethnic minority have been reported to be a barrier to accessing medication or services [1, 5, 11, 12]. However, the evidence base is limited in that studies examining what predicts medication use do not typically adjust for ADHD symptom severity, meaning factors that act as barriers to medication, but which are also associated with symptom severity are conflated in the findings.

The current study explores child, parent, and sociodemographic predictors of medication use among children with ADHD in a UK population-representative cohort. Our aims were to estimate the proportion of children with ADHD in the UK who are treated with medication, and to examine barriers to and predictors of medication use for ADHD at age 14, controlling for ADHD symptom severity at age seven.

## Methods

### Study design and sample characteristics

The Millennium Cohort Study (MCS) is a longitudinal UK population-representative cohort of British children born between the 1st September 2000 and the 11th January 2002. Families were eligible if they received child benefit (a universal benefit with near 100% uptake at the time). Detail on MCS design and sampling is reported extensively elsewhere [13–15]. The total number of families in the MCS was 19,244: the number responding at age 14 was 11,726. Weightings supplied by MCS are used to adjust for attrition, maintaining a sample representative of the UK population as a whole (see Missing Data).

Six waves of data collection have been conducted: when children were aged 9 months, 3, 5, 7, 11, and 14 years. At each wave information on a range of social, economic, and health topics was collected through structured interviews at the family home, and on occasion via teachers in schools. Cognitive assessments and other data were also collected.

All participants of the MCS provided informed consent and the main study had full ethical approval in place. Ethical approval for the current analysis was given by the University of Exeter College of Social Sciences and International Studies Ethics Committee (ref 201718-093).

## Measures

**Child ADHD** (yes/no). Parents were asked “*Has a doctor or health professional ever told you that [child’s name] has had any of the following problems: attention deficit/hyperactivity disorder (ADHD)?*” The child was classed as having ADHD if the parent responded in the affirmative at when the child was aged seven, 11 or 14. This question was based on the wording used by US National Health Interview Survey (NHIS) and National Survey of Children’s Health, which has been widely used to estimate prevalence of ADHD [5, 16] and Autism Spectrum Disorders [17].

**Outcome: ADHD medication** (yes/no). When children were age 14, parents who had reported their child had ADHD were asked “*Is [child’s name] currently taking any medicines on a regular basis that were prescribed by a doctor or hospital for their ADHD?*” A list of licensed ADHD medications was also provided, that included methylphenidate, dexamphetamine and atomoxetine.

**Age at medication initiation**: Those who indicated that their child was taking medication were asked “*How old was [child’s name] when [he/she] was first prescribed these medicines?*” Three responses of two years old and younger, and ‘don’t know’ were classified as missing as no ADHD medication is licensed for children under the age of three in the UK.

**ADHD symptom severity**: the Strengths and Difficulties Questionnaire (SDQ) [18]; a widely used and validated behavioural screening questionnaire, was completed by parents and teachers when the child was seven. The SDQ has a sensitivity of 89% and specificity of 78% for the subscale pertaining to ADHD in comparison with clinical diagnosis [19], and has satisfactory internal consistency (Chronbach’s α) of 0.49 in a UK sample [20]. The hyperactivity/inattention subscale (SDQ-H/I) is comprised of five questions distributed across the 25-item SDQ, and asks about the frequency of five behaviours common in ADHD covering inattention, impulsivity and hyperactivity. It is scored from 0 to 10 with higher scores indicating greater difficulties. Although the SDQ is more brief than other measures commonly used to assess ADHD, such as the Child Behaviour Checklist, it correlates highly with these more extensive measures [21]. An algorithm generating unlikely, possible and probable diagnosis of ADHD [19] was utilised to capture symptom severity: this combined both parent and teacher report of SDQ-H/I symptoms and their impact. This algorithm has been found to have a sensitivity of 75% for parent and teacher ratings combined [22] and has a high level of agreement with independent clinical diagnosis [19].

## Predictors of ADHD medication use

### Child-based characteristics

Predictors comprised child gender, cognitive ability at age three (score on the Bracken School Readiness Assessment, administered individually by computer [23]), and conduct problems. The SDQ conduct problems (SDQ-CP) score sums the responses from five items that ask about temper, anger and antisocial behaviour. Parent and teacher ratings of conduct problems at age seven were included as predictors of medication use: scores were treated as continuous with a range from 0 to 10. As conduct problems tend to occur later in childhood we also included parent-reported SDQ-CP at age 14.

### Parent characteristics

Mother report of ever being diagnosed with depression or anxiety at the child age seven wave (yes/no), and mother and father report of having a mental health or social/behavioural condition including autism or ADHD (yes/no) were included as predictors. Maternal age at child’s birth (years), and smoking during pregnancy (number of cigarettes per day) were also included.

### Sociodemographic characteristics

Characteristics measured at the 9 month wave were ethnicity (white British or other) and maternal education (no qualifications; secondary school; degree or higher). When the child was seven the remaining predictors were captured: family size (no siblings; one; two; or > two siblings), single parent family (vs two parents), family weekly income (in £, adjusted for number of adults and children in the household based on OECD scales for equivilisation), whether living in poverty (income ≤ 60% of the median household income for the UK population), and housing tenure in three categories (housing association; private rent; owned). We also provide descriptive statistics based on whether children were living in an urban or rural area at age 14, however, as this was calculated differently across countries in the UK we have not analysed this further.

### Analysis

The prevalence of ADHD by age 14 (parent-reported), overall and by gender was calculated relative to those who had responded in the negative to the ADHD diagnosis question. The proportion of children reported as having ADHD who used medication were described by gender and age of medication initiation. Descriptive statistics on predictors were generated showing the characteristics of children with medicated ADHD, non-medicated ADHD, and, for comparison, the wider MCS cohort.

Logistic regression models were used to calculate the association between child, socioeconomic and parent predictors and ADHD medication use. Our comparison was between non-medicated children with ADHD and those taking medication. Unadjusted bivariate associations, expressed as odds ratios (OR) and 95% confidence intervals (95% CI) were reported. Following this, variables that were associated with ADHD medication with a *p* value of ≤ 0.10 were carried forth into a multivariable regression model that adjusted for ADHD symptom severity. Continuous predictors (other than SDQ scores) were rescaled by dividing by two standard deviations, so that the strength of association was comparable across continuous and binary predictors [24]. ORs, therefore, indicated the relative increase in odds of taking medication for ADHD, corresponding to a two standard deviation increase in the predictor. All analyses were carried out in Stata v14.0 using the *svy* command and weights to adjust for sampling and attrition.

### Missing data

At age 14, only 0.09% of ADHD diagnosis data were missing, 1.3% of those who were asked the medication question did not provide a valid response. MCS study design accounted for attrition by over-sampling socioeconomically disadvantaged communities, then assessing drop-out at each time point. The MCS provides weights at each age point which account for drop-out in each sweep, keeping the sample representative of the UK population. Weights were generated using a logit model where the dependent variable was response/non-response, and included 12 variables predicting missingness. Weights were constructed and calculated based on the contribution of different sociodemographic predictors to this regression model [25]. As such these weightings account for missing data due to attrition. For the current analysis, weighted prevalence estimates were calculated and regression models also adopted the appropriate weightings.

As the SDQ algorithm data were not complete (teacher data were missing for around half the sample) we conducted a sensitivity analysis adjusting for parent-reported SDQ-H/I score at age seven. This measure has been used in other studies as a measure of ADHD symptoms [26].

## Results

Four hundred and seventy-one children were reported to have ADHD, which equated to a weighted prevalence of ADHD (*N* = 11,708) of 3.97% (95% CI 3.43, 4.60). Boys were five times more likely than girls to have ADHD (boys 6.41%, 95% CI 5.45, 7.52; girls 1.29%, 95% CI 0.96, 1.74); Table 1.

**Table 1:** Prevalence of parent-reported ADHD: overall and by gender

Sample	*n*	Weighted % with ADHD	95% Confidence interval
All	11,708	3.97	3.43, 4.60
Female	5839	1.29	0.96, 1.74
Male	5869	6.41	5.45, 7.52

**Fig. 1:**
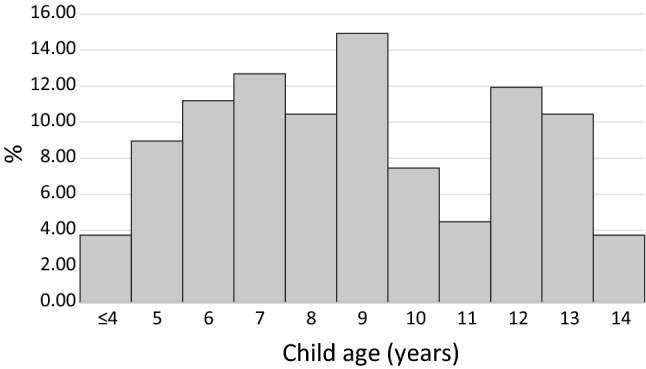
Age at medication initiation for children reported to be taking medication for ADHD

305 parents of children with ADHD provided valid responses to the medication question at age 14. Under half (45.6%) of children with ADHD were taking medication at this age (*N* = 139); of these 11.5% were female and 88.5% male. The median age that medication was reported to have been first taken was 9 years old (range 3–14). Most reported beginning to take medication during their primary school years (Fig. 1).

Table 2 shows characteristics of the sample both for children with ADHD with and without medication, and for the wider cohort. Children with ADHD were more often male, had younger mothers, had lower SES and a higher proportion of their parents reported mental health or social/behavioural conditions (based on *t* tests for continuous or chi^2^ for categorical variables). There were insufficient responses from fathers about these conditions to allow analysis beyond a descriptive level. Children with ADHD displayed higher SDQ-CP and SDQ-H/I scores than children in the wider cohort. Further, children with ADHD who received medication were more likely to have higher parent-report SDQ-H/I scores at age seven than their ADHD peers who did not, although these symptoms were not rated as significantly more impactful. This resulted in a higher proportion of children with ADHD who took medication being classified as ‘probable ADHD’ by the ADHD symptom algorithm than those who did not.

**Table 2:** Descriptive statistics for ADHD children with and without medication, compared to a population sample

	Age (years)	ADHD no medication (*N* = 166)		ADHD with medication (*N* = 139)		Rest of cohort (*N* = 18,938)	
		*n*		*n*		*n*	
**Child characteristics**							
Male (%)	< 1	166	78.31	139	88.49	18,938	50.91
Cognitive ability, mean (SD)	3	129	43.26 (32.00)	108	45.68 (30.08)	13,604	56.81 (30.50)
SDQ-CP: parent, mean (SD)	7	141	2.77 (2.09)	121	3.86 (2.25)	13,131	1.36 (1.51)
SDQ-CP: teacher, mean (SD)	7	85	2.11 (2.08)	84	2.86 (2.33)	8562	0.74 (1.45)
SDQ-CP: parent, mean (SD)	14	159	3.22 (2.51)	137	4.44 (2.51)	11,032	1.35 (1.54)
**Parent characteristics**							
Maternal age in years at childbirth, mean (SD)	< 1	156	26.90 (6.01)	133	26.31 (5.66)	18,125	28.33 (5.96)
Number of cigarettes smoked daily in pregnancy, mean (SD)	< 1	157	3.22 (5.87)	133	5.35 (8.75)	18,241	2.00 (4.99)
Maternal depression/anxiety (%)	7	63	7.94	56	14.29	8439	6.61
Maternal mental health condition (% yes)	14	63	41.27	59	45.76	2667	29.36
Maternal social/behavioural condition, e.g., autism ADHD (% yes)	14	63	4.76	59	8.47	2667	2.70
Paternal mental health condition (% yes)	14	21	19.05	19	26.32	1461	14.78
Paternal social/behavioural condition, e.g., autism ADHD (% yes)	14	21	4.76	19	15.79	1461	2.67
**Sociodemographic characteristics**							
White British (%)	< 1	156	84.62	133	93.98	18,080	82.49
Maternal education							
*No qualifications (%)*	< 1	156	20.51	134	27.61	18,194	19.46
*Secondary school (%)*			60.26		61.19		56.27
*Degree or higher (%)*			19.23		11.19		24.28
Family size							
*Only child (%)*	7	146	15.07	126	13.49	13,585	12.38
*1 Sibling (%)*			38.36		41.27		44.84
*2 Siblings (%)*			28.77		24.60		27.24
*More than 2 siblings (%)*			17.81		20.63		15.55
Single parent family (%)	7	146	33.56	126	31.75	13,585	20.82
Family weekly income in £, mean (SD)	7	144	346.03 (230.15)	125	311.535 (192.78)	13,398	382.67 (227.60)
Below poverty line (%)	7	146	42.47	126	42.86	13,565	29.83
Housing tenure							
*Social housing (%)*	7	141	39.01	121	42.15	13,140	23.01
*Rent private (%)*			15.6		12.4		8.76
*Home owner (%)*			45.39		45.45		68.23
Urban dwelling (%)	14	126	90.48	146	89.04	13,583	86.91
**Covariates**							
SDQ-HI parent (/10)	*7*	140	6.37 (2.96)	122	7.61 (2.44)	13,081	3.04 (2.47)
SDQ-HI teacher (/10)	*7*	85	6.35 (2.99)	84	7.04 (2.61)	8560	2.81 (2.76)
SDQ impact parent (/10)	*7*	141	1.97 (2.75)	121	2.58 (2.85)	12,978	0.27 (0.98)
SDQ impact teacher (/10)	*7*	68	1.78 (1.63)	71	2.25 (1.42)	7459	0.41 (0.97)
ADHD severity	*7*						
* Unlikely ADHD (%)*		141	48.23	125	33.60	13,162	91.64
* Possible ADHD (%)*			39.72		42.40		7.04
* Probable ADHD (%)*			12.06		24.00		1.31

Note: Age (years) refers to child age when data were collected: < 1 is the 9-month wave. ADHD: attention deficit/hyperactivity disorder. SD: standard deviation. SDQ: Strengths and Difficulties Questionnaire. CP: conduct problems subscale. HI: hyperactivity/inattention subscale ADHD severity at age 7 was defined by the SDQ ADHD algorithm, including parent and teacher symptom and impact reports. Urban–rural dwelling based on office for national statistics codes (England and Wales), the Birkbeck urban–rural indicator (Northern Ireland) and the Scottish Executive urban–rural classification (Scotland)

Predictors of medication use for children with ADHD at age 14 are shown in Table 3. Children who were taking medication were significantly more likely to be male (OR 3.37, 95% CI 1.57, 7.23, *p* = 0.002), have higher levels of conduct problems at age seven (SDQ-CP parent OR 1.26, 95% CI 1.09, 1.47, *p* = 0.002, SDQ-CP teacher OR 1.20, 95% CI 1.02, 1.42, *p* = 0.03) and age 14 (OR 1.20, 95% CI 1.05, 1.35, *p* = 0.003) and have mothers who reported smoking more cigarettes when pregnant (OR 1.60, 95% CI 1.10, 2.31, *p* = 0.01) in unadjusted regression models. White British ethnicity was associated with ADHD medication at *p* < 0.10, so this was taken forward into the adjusted model. After adjustment for symptom severity at age seven, male gender and parent-reported conduct problems at age seven and 14 remained significantly associated with medication use at age 14 (see Table 3). Males were more than three times as likely to receive medication. Each one point increase on the SDQ-CP corresponded to a 24% increase in likelihood of ADHD medication at age 14. When repeating the adjusted models with the parent SDQ-H/I as an indicator of symptom severity instead of the SDQ algorithm, our findings remained almost identical (data available from lead author on request).

**Table 3:** Unadjusted and adjusted logistic regression models exploring predictors of ADHD medication use

	Unadjusted				Adjusted for age 7 ADHD severity			
	*n*	OR	95% CI	*p*	*n*	OR	95% CI	*p*
**Child characteristics**								
Male	305	3.37	1.57, 7.23	0.002	266	3.66	1.75, 7.66	0.001
Cognitive ability	237	1.41	0.77, 2.58	0.26				
SDQ-CP: parent age 7	262	1.26	1.09, 1.47	0.002	262	1.24	1.04, 1.47	0.015
SDQ-CP: teacher age 7	169	1.20	1.02, 1.42	0.031	169	1.19	0.98, 1.45	0.080
SDQ-CP: parent age 14	296	1.20	1.07, 1.36	0.003	259	1.19	1.05, 1.35	0.006
**Parent characteristics**								
Maternal age in years at childbirth	289	0.88	0.49, 1.58	0.66				
Number of cigarettes smoked daily in pregnancy	158	2.35	1.14, 4.84	0.02	253	1.45	0.95, 2.23	0.09
Maternal depression/anxiety	119	3.20	0.72, 14.20	0.12				
Maternal mental health condition	122	1.37	0.57, 3.30	0.47				
Maternal social/behavioural condition, e.g., autism ADHD	122	1.58	0.38, 6.59	0.53				
**Sociodemographic characteristics**								
White British	289	2.68	0.98, 7.30	0.06	252	3.04	0.78, 11.77	0.11
Maternal education	290							
*No qualifications*		ref						
*Secondary school*		0.79	0.46, 1.36	0.39				
*Degree or higher*		0.64	0.27, 1.53	0.32				
Family size								
*Only child*	272	Ref						
*1 Sibling*		1.08	0.47, 2.47	0.86				
*2 Siblings*		0.81	0.33, 1.97	0.64				
*More than 2 siblings*		1.21	0.45, 3.22	0.70				
Single parent family	272	0.92	0.53, 1.62	0.78				
Family weekly income in £	269	0.68	0.38, 1.22	0.19				
Below poverty line	272	1.14	0.63, 2.04	0.67				
Housing tenure								
*Social housing*	262	Ref						
*Rent private*		0.60	0.23, 1.58	0.30				
*Home owner*		0.85	0.48, 1.51	0.57				

Note: ADHD severity at age 7 was defined by the SDQ probable ADHD algorithm including parent and teacher symptom and impact reports. Predictors significant in unadjusted analysis were carried forward to the adjusted model. *ADHD* attention deficit/hyperactivity disorder, *OR* odds ratio, *CI* confidence interval, *SDQ-CP* Strengths and Difficulties Questionnaire, conduct problems subscale

## Discussion

Our study is the first we know of to assess predictors of medication whist adjusting for ADHD symptom severity. Girls with ADHD were less likely to be prescribed medication even when they display similar ADHD symptom levels to boys. Conduct problems also predicted medication independently of ADHD symptoms, at both age seven and 14. Our findings show that these factors are important predictors of ADHD medication, even when symptom severity is accounted for.

Existing studies have reported that 36–43% of children with ADHD in the UK are prescribed medication [1, 27, 28]: lower than rates reported in Europe that range from 43 to 67% [7, 10, 29]. Recent analysis conducted in the USA with similar methodology to the study we report here found that 62% of 2–17 year olds with current ADHD were taking medication in 2016 [5]. Rates of medication use in Canada have recently been estimated at 70% for those aged up to 24 [6], and in East Asia 62% of young people with a new diagnosis of ADHD initiated pharmacological treatment in a 1-year observational study [9]. Existing studies that previous UK estimates derive from use small samples, primary care data or are over 10 years old: as such there is a need for further research in this area, especially given concern in the scientific literature and media about prescription rates for ADHD [30]. In the current study, 45.6% of children with ADHD were reported to be taking medication at age 14. The proportion of children with ADHD using medication remains lower than in North America, East Asia, France and Central Europe and in line with recent statistics from Germany (43.1% in 14 year olds in 2014) [5–7, 9, 10, 29].

As our data include only those who were taking medication at age 14 this may be an underestimate of the true prevalence of medicated ADHD as we would not capture those who discontinued prior to age 14. Having said this, prevalence of medication use for childhood ADHD has been reported to peak at age 13–14 [29]: it will be of interest to explore whether the proportion of children in the MCS using medication subsequently decreases.

There were three peaks in age of medication initiation: at ages seven, nine and 12. At age seven, children in UK schools are subject to the first national tests. Consequently, there are increasing demands on cognition, behaviour and attention with children expected to remain seated and focussed [31]. The peak at age 12 corresponds to recently entering secondary school. The social and organisational demands of secondary schools are a large ‘jump’ from primary schools: children are expected to organise and bring their own study materials, and move from class to class throughout the day. It is likely that children with ADHD who could cope with demands at primary school level, where staff and location are consistent, may struggle more than their peers upon entry to secondary school [32] and medication may, therefore, become a viable option to enable children to function at their best: indeed one of the major long term impacts of ADHD is poor educational attainment [33]. In addition at age 12 children are entering adolescence, and physical, emotional and mental changes associated with this may be linked to the initiation of medication.

We found medication was initiated at age three and four in a small number of children (4% of those who received medication). A larger proportion received medication at age five. Previous UK reports have been limited to children age six and above [34]. Early medication of children has been a source of concern for ethicists [35] who have argued that very young children typically show inattention and hyperactivity [36], thus it may not be appropriate. Alternately, it may be that children who are prescribed medication at such a young age comprise a group with severe symptoms that has led to family stress or exclusion from child care. Further research should explore this.

Many studies have found that more severe ADHD is associated with medication use [7, 9, 10, 27]. Gender also predicts more severe symptoms of ADHD [30]. We found that symptom severity was a powerful predictor of ADHD medication, with children who took medication suffering from more severe symptomology than those who did not. This finding aligns with clinical guidelines that suggest non-pharmacological treatment should be the first line of treatment for ADHD [37], so we would expect medication to be prescribed only for more severe cases.

Taking symptom severity into account, boys were over three times more likely to be taking medication for their ADHD than girls: a finding not seen in some other studies [5–7]. Treatment choice and decisions may be influenced by gender bias in beliefs about how likely the child is to benefit from the treatment, and stigma operating differentially by child gender [4]. It could be that girls are perceived by parents, teachers or clinicians as less likely to benefit from pharmacological treatment or more likely to benefit from behavioural treatments: evidence in this area is lacking. Our study provides evidence that even if recognised, girls may face additional barriers to access treatment: clinicians should be aware of this. Alternatively, some have argued ADHD is too frequently diagnosed in boys, and that clinicians think a prototypical ADHD child is male. This might lead to boys being more often recognised and treated [38].

The second significant predictor of whether children received ADHD medication was conduct problems. The SDQ-CP subscale asks about temper, obedience, fighting, lying and stealing. Our finding is in line with other studies that report co-occurring conduct symptoms to be more frequent in those who receive medication for ADHD [5, 7, 8, 10]. This raises debate as to whether medication for ADHD is being prescribed to those who display problematic behaviour beyond ADHD, or whether those with the most severe or complex behavioural difficulties are taking medication because of this complexity. A moderator analysis of the multimodal treatment for ADHD study found that children with comorbid conduct or oppositional defiant disorder at baseline responded similarly to treatment to those without these comorbidities [39]: medication was not disproportionately beneficial to these children. However, a recent systematic review reports evidence that both psychostimulants and atomoxetine are effective in reducing disruptive and aggressive behaviours in children with ADHD [40]. Our findings raise questions about diagnostic silos and whether children with both ADHD and conduct disorder should be viewed through the ADHD or conduct disorder lens. If medications currently licensed to treat ADHD are beneficial for a primary diagnosis of conduct disorder, consideration of whether UK clinical guidelines and licensing should be changed is needed. The National Institute for Health and Care Excellence (NICE) ADHD guideline recommends non-pharmacological treatments as the first option for those with ADHD and symptoms of conduct disorder [37], however, guidelines from Canada and other countries recommend medication in these cases (e.g., [41, 42]). Clinicians should be aware that severity of problems in comorbid ADHD and conduct disorder may be driven by the latter. Monitoring treatment response is, therefore, essential to ensure medication is beneficial in these cases.

Although children with ADHD were of lower SES than the MCS cohort, no measure of SES was associated with ADHD medication use. This is encouraging as it suggests that the current system is free from bias in either direction: it has been argued that low SES may preclude individuals from medication access because of the affordability of medication, or stigma around the perceived cause of the child’s symptoms. As the UK healthcare system is free at the point of use, and prescriptions are free for those on low incomes, our results may reflect societal organisational structures that allow healthcare access equally to those from different socioeconomic backgrounds.

Previous studies [11, 43], found an association between medication and socioeconomic disadvantage, but reported opposing directions of effect. The study by Froehlich et al. using national survey data in the USA found low SES to be a barrier to medication use, whereas the Swedish register-based study of Hjern et al. finds socioeconomic disadvantage to be a strong predictor of ADHD medication use. There appears to be an interaction between ADHD being more prevalent and severe in those who grow up in socioeconomically disadvantaged households [44] and the healthcare system in the country where the study is conducted. Our findings highlight that each healthcare system will have its own biases in prescribing practices and throw the status of low SES as a barrier to medication into question.

## Strengths and limitations

The use of longitudinal cohort data is necessarily constrained by the range of data collected. Cohorts are not best suited for estimating prevalence due to the inevitable attrition, although to an extent this is statistically accounted for by the sampling frame and weighting. Our case definition of ADHD was parent-report of whether they have been told that their child has ADHD, and omits precise terminology about diagnosis. Parent-report of ADHD diagnosis using terminology almost identical to that used in the MCS is considered appropriate for national estimates of ADHD prevalence in the USA [45]. The validity of parent-report of child medication for ADHD has been shown to be good, with > 80% agreement between parent-report and clinical records [46]. Studies that examine the validity of parent-reported diagnosis are lacking, however.

We are unable to ascertain whether children who received a diagnosis of ADHD had this rescinded prior to age 14: our estimates are therefore of ever receiving a diagnosis, rather than current diagnosis at 14. Medication use and age of medication initiation was restricted to those who were currently taking medication at the age of 14. Other children with ADHD may have used medication but discontinued this by the age of 14, or received a diagnosis of ADHD that did not persist to age 14. Despite these limitations, the proportion of children reported to have ADHD in MCS falls in line with current prevalence estimates [47]. A further limitation is that data on the type of medication being used was not collected.

The study also has strengths: a population-representative sample that has over 300 children with ADHD with pertinent longitudinal data, collected relatively recently. There were some missing data: parent mental health questions tended to be the least frequently answered (perhaps due to their sensitive nature), and teacher SDQs were not completed for all children. However, we considered it important to have a measure of symptom severity that did not solely rely on a parent informant, as ADHD must be present across settings, and conducted a sensitivity analysis using parent SDQ-H/I scores. Due to small sample sizes and occurrence of missing data, we did not have adequate power to explore whether some characteristics (such as paternal ADHD) were predictive of child ADHD medication use.

More work is required into whether children with conduct disorders are appropriately receiving medication for comorbid ADHD. Further exploration of predictors of medication use by medication type, and whether treatment response is associated with any of these predictors is needed. Finally, further research should explore the other treatment options that children are being offered to manage their ADHD (e.g., [9]) as we are unable to ascertain what proportion of children were untreated or treated using psychosocial therapies.

## Data Availability

The MCS data is freely available to accredited researchers via the UK Data Service. https://www.ukdataservice.ac.uk/.
